# Demographic and clinical factors associated with recovery of poststroke dysphagia: A meta‐analysis

**DOI:** 10.1002/brb3.3033

**Published:** 2023-05-15

**Authors:** Lian Wang, Jia Qiao, Fang Sun, Xiaomei Wei, Zulin Dou

**Affiliations:** ^1^ Department of Rehabilitation Medicine The Third Affiliated Hospital of Sun Yat‐sen University Guangzhou China; ^2^ Clinical Medical of Acupuncture Moxibustion and Rehabilitation Guangzhou University of Chinese Medicine Guangzhou China

**Keywords:** dysphagia, factors, meta‐analysis, prognosis, stroke

## Abstract

**Background:**

Poststroke dysphagia (PSD) recovery depends on various factors. We aimed to provide evidence concerning predictive variables for the recovery of PSD.

**Methods:**

PubMed, Embase, Web of Science, China National Knowledge Infrastructure (CNKI), Wanfang Database, VIP database of Chinese periodicals, Chinese biomedical literature service system (SinoMed), and Cochrane Library databases were systematically searched up to September 21, 2022. According to the inclusion criteria, the literature searched in the database was screened. The methodological quality of included studies was assessed using the Newcastle‐Ottawa Scale (NOS). Meta‐analysis was performed to identify the factors prognostic for PSD.

**Results:**

Twenty‐eight studies were eligible, and pooled analyses were allowed for 12 potential prognostic factors. We identified older age, higher National Institutes of Health Stroke Scale (NIHSS) score, lower activities of daily living (ADL) score, lower body mass index (BMI), severe dysphagia on admission, aspiration, brainstem stroke, severe cognitive impairment, and bilateral hemispheric stroke were negative factors for the recovery of PSD, while early intervention and Modified Rankin Scale (mRS) = 0 before onset were protective factors for the recovery of PSD. There was no significant association between stroke type and prognosis of PSD.

**Conclusion:**

Prognostic factors of PSD summarized in this meta‐analysis could be useful for developing reasonable treatment plan to better promote recovery of swallowing function after stroke.

## Graphical abstract

Dysphagia, as one of the common sequelae after stroke, has a devastating impact on patients. There are several factors influencing the prognosis of post‐stroke dysphagia (PSD). Studies have been conducted to show that several demographic and clinical factors are associated with the prognosis of PSD, but a summary of these factors is lacking. This meta‐analysis summarizes the association between relevant factors and the prognosis of PSD by pooling multiple studies, which is crucial for further determining individualized treatment to promote patient recovery.

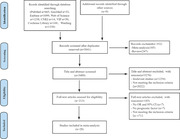

## 1 INTRODUCTION

Stroke is a major cause of disability globally, leading to different aspects of functional impairment (Cambell & Khatri, 2020, Kumar et al., 2010). Dysphagia as one of the common sequelae after stroke has been shown to be associated with the increased risk of aspiration, pneumonia, and malnutrition, which in turn further impedes the patients’ return to premorbid function (Cohen et al., 2016). Moreover, dysphagia has an adverse impact on the long‐term quality of life not only for the patient but also for their family members or caregivers, particularly in the event that the patient undergoes a percutaneous endoscopic gastrostomy (Mori et al., 2019). Even though, dysphagia usually improves spontaneously and quickly, the problems could persist for 6 months or even longer in approximately 10% of patients (Dawson et al., 2016; Smithard et al., 1997; Takahata et al., 2011). In light of this, assessing the prognosis of poststroke dysphagia (PSD) to optimize therapeutic protocols for better functional recovery of stroke patients is absolutely necessary. However, methods to assess the prognosis of PSD have not been well developed.

The trajectory of recovery from PSD varies depending on various factors. Currently, assessment of PSD prognosis based on predictive variables is becoming a feasible approach. Several studies have identified some factors that could potentially affect the recovery of dysphagia in stroke survivors, including older age (Dubin et al., 2013), body mass index (BMI) (Ikenaga et al., 2017), signs of aspiration (Ickenstein et al., 2012), the National Institutes of Health Stroke Scale (NIHSS) (Galovic et al., 2013), stroke type (Inooka et al., 2022), bihemispheric lesions (Kumar et al., 2012), and intubation (Kumar et al., 2014). However, these findings were inconsistent and not definitive, and reliable predictors of PSD have not yet been clearly concluded.

In order to develop individualized therapeutic programs and to provide patients with a reasonable anticipation of outcome, factors associated with recovery of PSD deserve attention. Therefore, the primary purpose of this meta‐analysis was to summarize the association between relevant variables and the prognosis of PSD.

## 2 METHODS

This study was conducted in compliance with the Preferred Reporting Items for Systematic Reviews and Meta‐Analyses (PRISMA) reporting guidelines. A protocol was registered in the PROSPERO database (CRD42022369679).

### 2.1 Search strategy and eligibility criteria

We conducted a systematic search of PubMed, Embase, Web of Science, China National Knowledge Infrastructure (CNKI), Wanfang Database, VIP database of Chinese periodicals, Chinese biomedical literature service system (SinoMed), and Cochrane Library databases. Searches were performed without language of publication restriction, and the search time frame was from the establishment of each database to September 21, 2022. The full search strategy was detailed in Supplementary.

Studies included met the following criteria: (1) case‐control or cohort studies investigating factors that contribute to the prognosis of PSD; (2) subjects enrolled in the study were adults (aged ≥18 years); (3) data including odds ratio (OR) and 95% confidence interval (CI) can be extracted from the manuscript; (4) studies published in English or Chinese. Case reports, conference abstracts, letters, review articles, and studies from which relevant data cannot be extracted were excluded.

### 2.2 Data extraction and quality assessment

Retrieved records from database searches were imported into NoteExpress and duplicate records were removed. Two independent reviewers screened the paper for title, abstract, and full text. When discrepancies arose during the selection process, two other reviewers were consulted to determine whether the literature fulfilled criteria. The following data were extracted from all eligible studies: first author, year of publication, country, study design, sample size, prognostic risks, and odds ratio (OR) and 95% confidence interval (CI) for variables of interest. Data extraction was performed independently by two well‐trained reviewers and filled into a predefined Excel sheet (Microsoft).

The methodological quality of included studies was assessed using the Newcastle‐Ottawa Scale (NOS), which was developed to provide an assessment of case‐control and cohort studies and includes eight items in three different domains with a maximum quality of 9 stars, and the more stars, the lower the risk of bias. Studies with 1–3 stars were considered low quality, studies with 4–6 stars were considered medium quality, and those with greater than 7 stars were considered high quality.

### 2.3 Statistical analysis

Analyses were conducted for prognostic factor which were involved in at least three studies. The OR and 95% CI for poststroke dysphagia were pooled in a meta‐analysis using RevMan Software 5.1.

Heterogeneity was examined using Cochran Q, and degrees of heterogeneity were quantified using the *I* ^2^ statistic. Cochran Q *p* < .10 or *I* ^2^ >50% was considered to reflect significant heterogeneity. Sensitivity analyses were performed by removing individual study each time to explore the impact of each study on the overall risk estimate. Based on heterogeneity, random‐effects models and fixed‐effects models were used in the meta‐analysis to analyze the data as appropriate. Potential publication bias was assessed quantitatively using Stata/SE 15.1 with Begg's test and Egger's test. Statistical significance was set as *p* < .05.

## 3 RESULTS

### 3.1 Search results

**FIGURE 1:**
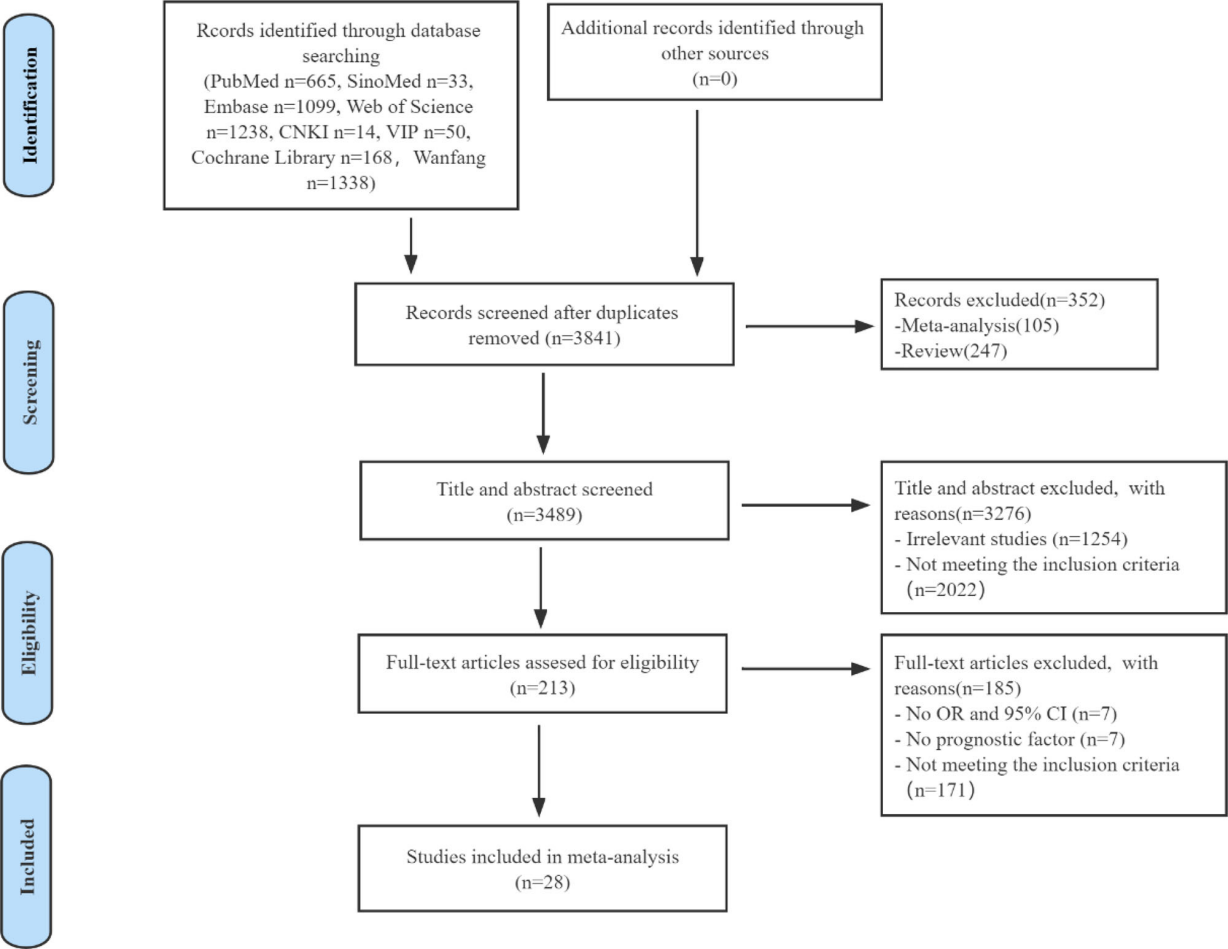
PRISMA flowchart.

Using the research protocol, 4605 relevant studies were retrieved from the databases, of which 764 were excluded for duplication and 3276 were excluded following title and abstract screening. Finally, 28 publications (Calvo et al., 2019; Hu et al., 2013; Ikenaga et al., 2017; Inooka et al., 2022; Kumar et al., 2014; Lan et al., 2002; Liao et al., 2022; Lin et al., 2019; Myung & Pyun, 2022; Nakadate et al., 2016; Nakajima et al., 2012; Nakajima et al., 2012; Nishioka et al., 2017; Ogawa et al., 2021; Peng & Yuan, 2006; Shimizu et al., 2019; Sreedharan et al., 2022; Takahata et al., 2011; Toscano et al., 2015; Wang et al., 2022; Wang et al., 2011; Wang et al., 2022; Wei et al., 2010; Xi et al., 2021; Xie et al., 2015; Zhan & Jiang, 2018; Zhang et al., 2022; Zhang et al., 2012) were included after reading the full text. Figure 1 shows the flow diagram of studies selection process.

Of the 28 studies included, there were 27 cohort studies and 1 case‐control study. A total of 14 studies were conducted in China, 9 in Japan, 2 in Italy, 1 in Korea, 1 in America, and 1 in India (Table 1).

**TABLE 1:** Characteristics of the included studies

Study	Publication year	Country	Study design	Sample size	Setting	Swallowing assessment methods	Prognostic factor	NOS score
Nakajima et al.	2012	Japan	Cohort	512	Hospital	WST/SST	1, 2, 11, 38, 44	7
Nakadate et al.	2016	Japan	Cohort	107	Hospital	VFSS	1, 4, 14	8
Wang et al.	2022	China	Cohort	485	Hospital	WST/VFSS/FEES	1, 2, 4, 6, 9, 10	9
Inooka et al.	2022	Japan	Cohort	151,302	Hospital	Claims record for diet	1, 4, 11, 12, 27, 28	7
Liao et al.	2022	China	Cohort	234	Hospital	WST	1, 2, 3, 46	9
Zhang et al.	2012	China	Cohort	179	Hospital	SSA	1, 3, 30	9
Lan et al.	2002	China	Cohort	56	Hospital	CED	1, 3, 9, 47	8
Wang et al.	2011	China	Cohort	116	Hospital	WST	1, 3	9
Nakajima M et al.	2012	Japan	Cohort	525	Hospital	WST /RSST	1, 2, 11, 32	8
Calvo et al.	2019	Italy	Cohort	163	Hospital	BLB	1, 7, 27, 33	9
Zhan et al.	2018	China	Cohort	170	Hospital	WST	1, 9, 10, 13	8
Xie et al.	2015	China	Cohort	296	Hospital	SSA	1, 2, 3, 5, 7, 36	7
Shimizu et al.	2019	Japan	Cohort	188	Hospital	IDDSI‐FDS	1, 3, 6, 8	8
Takahata et al.	2011	Japan	Cohort	219	Hospital	Foodtest/FESS/VFSS	1, 5, 28, 43, 45	8
Zhang et al.	2022	China	Cohort	51	Hospital	WST	2, 15, 19	8
Sreedharan et al.	2022	India	Cohort	469	Hospital	GUSS	2, 11, 15, 24, 25	8
Wang et al.	2022	China	Cohort	141	Hospital	WST/VFSS	2, 16, 17	8
Kumar et al.	2012	America	Cohort	323	Hospital	Bedside swallowing evaluation/VFSS	2, 7, 10, 19, 29, 34	8
Toscano et al.	2015	Italy	Cohort	254	Hospital	WST	2, 12, 13, 37	8
Ikenaga et al.	2017	Japan	Case‐control	72	Hospital	VFSS/FEES	4, 6, 13	8
Peng et al.	2006	China	Cohort	84	Hospital	COSF	5, 6, 13, 31	9
Nishioka et al.	2016	Japan	Cohort	264	Hospital	FSG	3, 5, 11, 8, 35	8
Hu et al.	2013	China	Cohort	80	Hospital	COSF	5, 6, 13, 31	8
Ogawa et al.	2021	Japan	Cohort	274	Hospital	RSST/WST	5, 6, 40	7
Xi et al.	2021	China	Cohort	180	Hospital	WST	12, 13, 18, 41	8
Wei et al.	2010	China	Cohort	118	Hospital	WST	13, 16, 20, 21, 22, 23, 39	8
Myung et al.	2022	Korea	Cohort	130	Hospital	VFSS	42	8
Lin et al.	2019	China	Cohort	165	Hospital	FOIS	22, 26	8

1. Age; 2. National Institutes of Health Stroke Scale (NIHSS) score; 3. Activities of daily living; 4. Body mass index; 5. Early intervention; 6. Severe dysphagia on admission; 7. Aspiration; 8. Malnutrition; 9. Brainstem stroke; 10. Bilateral hemispheric stroke; 11. Modified Rankin Scale (mRS) = 0 before onset; 12. Type of stroke; 13. Cognitive impairment; 14. Elevated white blood cell count; 15. Infarct site; 16. Autonomous cough; 17. Electrical stimulation; 18. Hemoglobin; 19. Dysarthria; 20. Tongue hemiplegia; 21. Difficulty with tongue uplift; 22. Facial paralysis; 23. Change in voice after eating; 24. Moderate‐severe dysphagia at discharge; 25. In hospital worsening; 26. Aphasia; 27. Sex; 28. Consciousness disorders; 29. Intubation; 30. Low‐density lipoprotein; 31. Visual and hearing impairment; 32. Atrial fibrillation; 33. Residues; 34. Length of hospitalization; 35. Pneumonia; 36. Tracheal intubation; 37. White matter impairment of the brain; 38. Absence of hyperlipidemia; 39. Loss of gag reflex; 40. Flexible endoscopic evaluation of swallowing examination within 48 h of admission; 41. Drooling severity scale; 42. Oral apraxia; 43. Hemorrhagic lesions; 44. Noncardioembolism; 45. Hematoma volume; 46. Muscle strength; 47. Multisite stroke.

WST, Water Swallow Test; RSST, Repetitive Saliva Swallowing Test; CED, Clinical Examination for Dysphagia; BLB, Bilancio Logopedico Breve.

SSA, Standardized Bedside Swallowing Assessment; IDDSI‐FDS, International Dysphagia Diet Standardization Initiative Functional Diet Scale.

GUSS, Gugging Swallow Screening; FSG, Fujishima's Swallowing Grade; FOIS, Functional Oral Intake Scale; COSF, Classification of Swallowing Function.

### 3.2 Prognostic factors

Based on included 28 studies, there were 47 prognostic factors and 12 of them were feasible for meta‐analysis. The overall quality of the included studies was assessed using the NOS tool, and all 28 studies were determined to be of high quality (Table 1). Results of heterogeneity and publication bias tests of the included studies were shown in Table 2.

**TABLE 2:** Results of the pooled analysis of predictors

Prognostic factor	Studies involved	Model	Heterogeneity		OR(95% CI)	Test for overall effect (*p* value)	Publication bias test	
			*p* value	(*I* ^2^)			Begg's test (*p*)	Egger's test (*p*)
Age	6	Fixed	.46	0%	1.06 (1.04, 1.09)	<.00001	.452	.098
NIHSS score	6	Fixed	.15	38%	3.47 (2.68, 4.49)	<.00001	.260	.133
BMI	3	Fixed	.83	0%	1.28 (1.17, 1.40)	<.00001	1.000	.455
Brainstem stroke	3	Fixed	.86	0%	5.06 (3.11, 8.25)	<.00001	1.000	.660
Bilateral hemispheric stroke	3	Fixed	.31	15%	3.10 (2.04, 4.72)	<.00001	1.000	.455
ADL	6	Random	**.04**	57%	1.70 (1.28, 2.26)	.0002	.452	.111
Aspiration	3	Fixed	.36	1%	4.87 (3.18, 7.45)	<.00001	1.000	.512
mRS = 0 before onset	3	Fixed	.54	0%	0.58 (0.47, 0.71)	<.00001	.296	.067
Cognitive impairment	3	Random	.13	51%	1.02 (1.00, 1.04)	.03	1.000	.263
Early intervention	4	Fixed	.30	17%	0.74 (0.70, 0.78)	<.00001	.089	.056
Severe dysphagia on admission	3	Fixed	.32	11%	1.11 (1.07, 1.15)	<.00001	.296	.111
Hemorrhagic stroke	3	Random	**.01**	78%	1.04 (0.37, 2.99)	.94	1.000	.991

NIHSS, National Institutes of Health Stroke Scale; BMI, body mass index; ADL, activities of daily living; mRS, Modified Rankin Scale.

### 3.3 Older age

**FIGURE 2:**
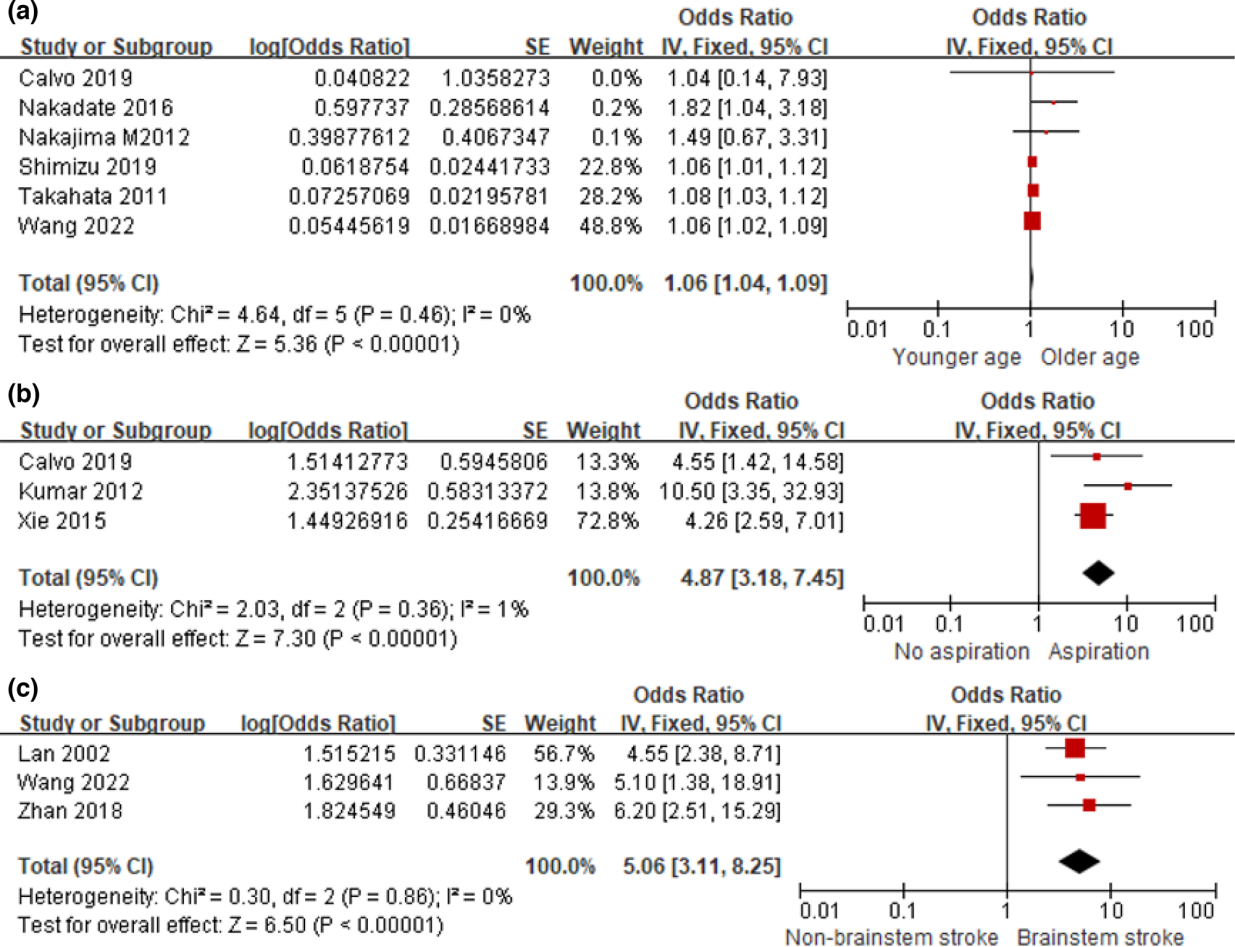
Subgroup analysis of prognostic risks. (**A**) Forest plot for age. (**B**) Forest plot for aspiration. (**C**) Forest plot for brainstem.

Older age as a risk factor for PSD was evaluated in 14 studies. Statistically significant heterogeneity was found among these 14 studies, and thus sensitivity analyses were performed and found that 8 studies contributed considerably to the heterogeneity, for which they were removed and meta‐analysis was performed using fixed‐effects model (*I* ^2^ = 0%; *p* = .46 > .10), with a pooled OR of 1.06 (95% CI, 1.04–1.09) (Figure 2A).

### 3.4 Aspiration

Three studies evaluated the impact of aspiration on the prognosis of PSD. The pooled OR from 3 studies did show a significantly increased risk (OR, 4.87; 95% CI, 3.18–7.45) under the fixed‐effects model (*I* ^2^ = 1%; *p* = .36 > .10) (Figure 2B).

### 3.5 Brainstem stroke

Pooled results from 3 studies showed that brainstem stroke is a risk factor for the recovery of dysphagia in stroke patients (OR, 5.06; 95% CI, 3.11–8.25; *I* ^2^ = 0%) (Figure 2C).

### 3.6 Bilateral hemispheric stroke

**FIGURE 3:**
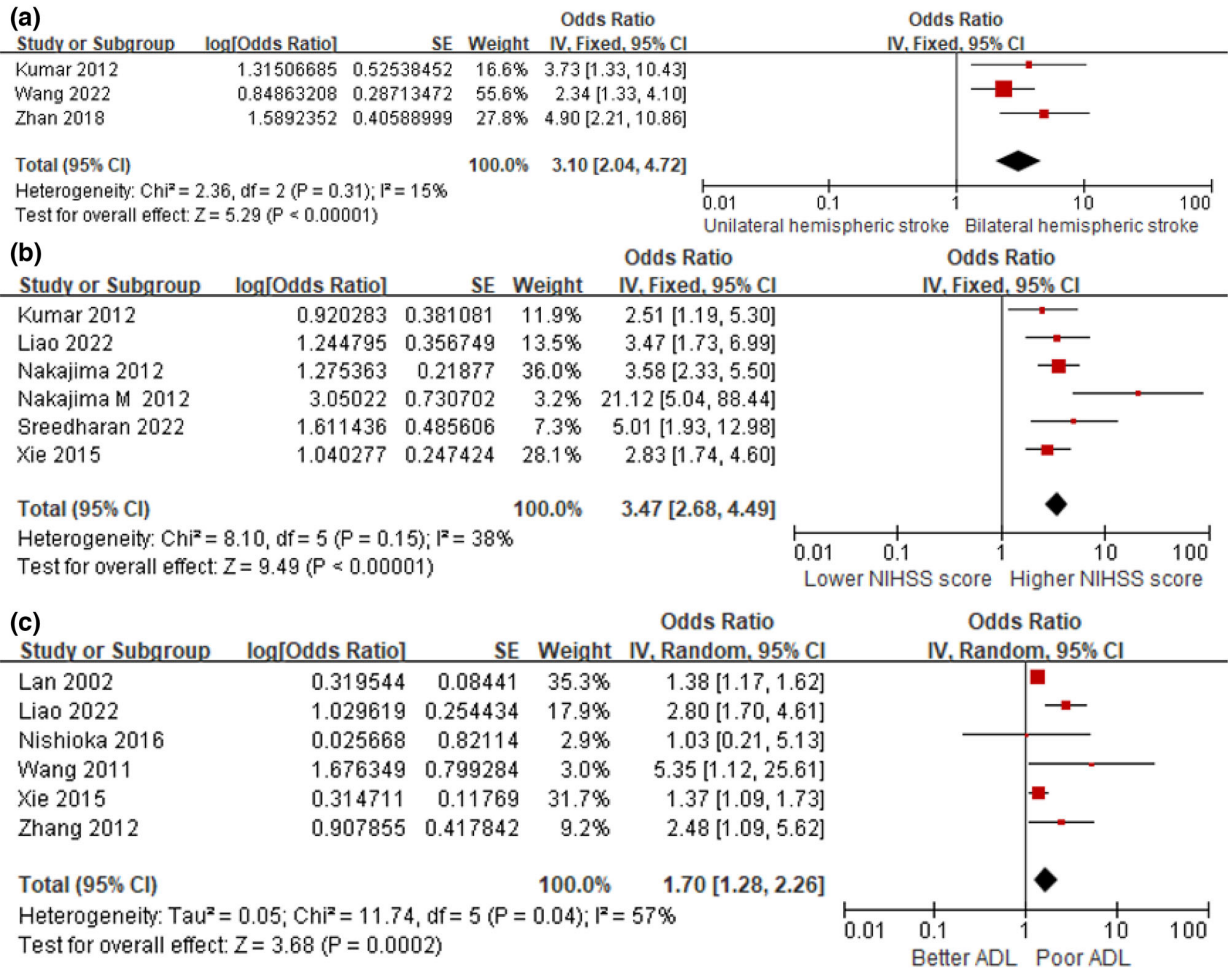
Subgroup analysis of prognostic risks. (**A**) Forest plot for bilateral hemispheric stroke. (**B**) Forest plot for NIHSS score. (**C**) Forest plot for ADL.

The impact of bilateral hemispheric stroke on the prognosis of PSD was investigated in 3 studies. The fixed‐effects model of 3 studies resulted in a pooled OR of 3.10 (95% CI, 2.04–4.72; *I* ^2^ = 15%) (Figure 3A).

### 3.7 NIHSS score

Of 10 studies assessing the relationship between NIHSS and the prognosis for PSD, due to significant heterogeneity, 6 studies were ultimately included after sensitivity analyses and with a pooled OR of 3.47 (95% CI, 2.68–4.49; *I* ^2^ = 38%) using fixed‐effects model (Figure 3B).

### 3.8 ADL

Patients who have poorer ability to perform daily activities are associated with a poorer prognosis for swallowing function, with a pooled OR of 1.70 (95% CI, 1.28–2.26; *I* ^2^ = 57%) (Figure 3C).

### 3.9 BMI

**FIGURE 4:**
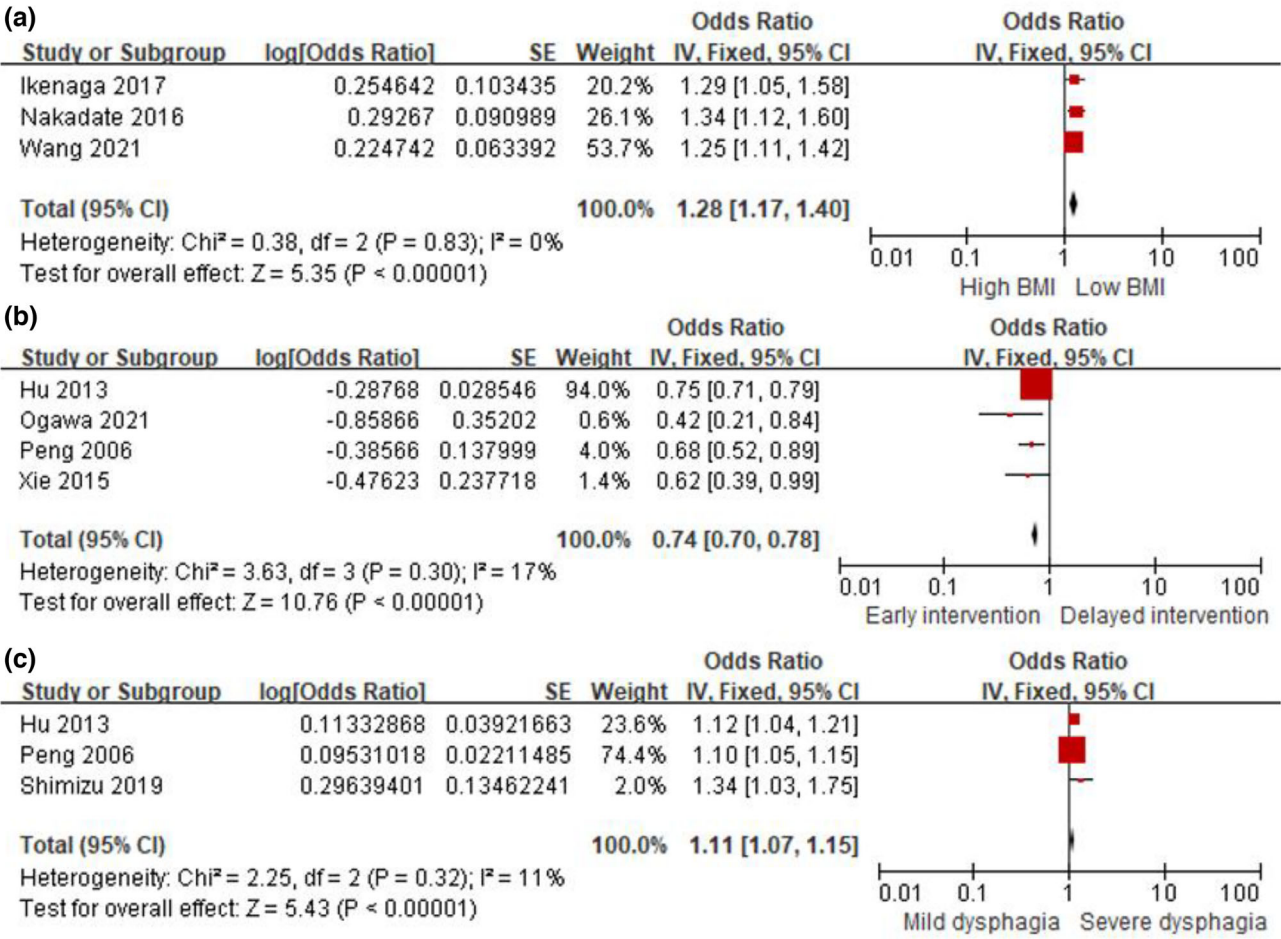
Subgroup analysis of prognostic risks. (**A**) Forest plot for BMI. (**B**) Forest plot for early intervention. (**C**) Forest plot for severity of dysphagia.

Four studies reported the association between BMI and the prognosis for PSD. The pooled OR of 3 studies was 1.28 (95% CI, 1.17–1.40; *I* ^2^ = 0%) (Figure 4A).

### 3.10 Early intervention

Using the random‐effects model, the pooled OR from 4 studies was 0.74 (95% CI, 0.70–0.78; *I* ^2^ = 17%), indicating that early intervention act as protective factors for prognosis of PSD (Figure 4B).

### 3.11 Severe dysphagia on admission

Of 6 studies evaluated the impact of severe dysphagia at admission on the prognosis of PSD, the pooled OR of 3 studies was 1.11 (95% CI, 1.07–1.15; *I* ^2^ = 11%) (Figure 4C).

### 3.12 Modified Rankin Scale (mRS) = 0 before onset

**FIGURE 5:**
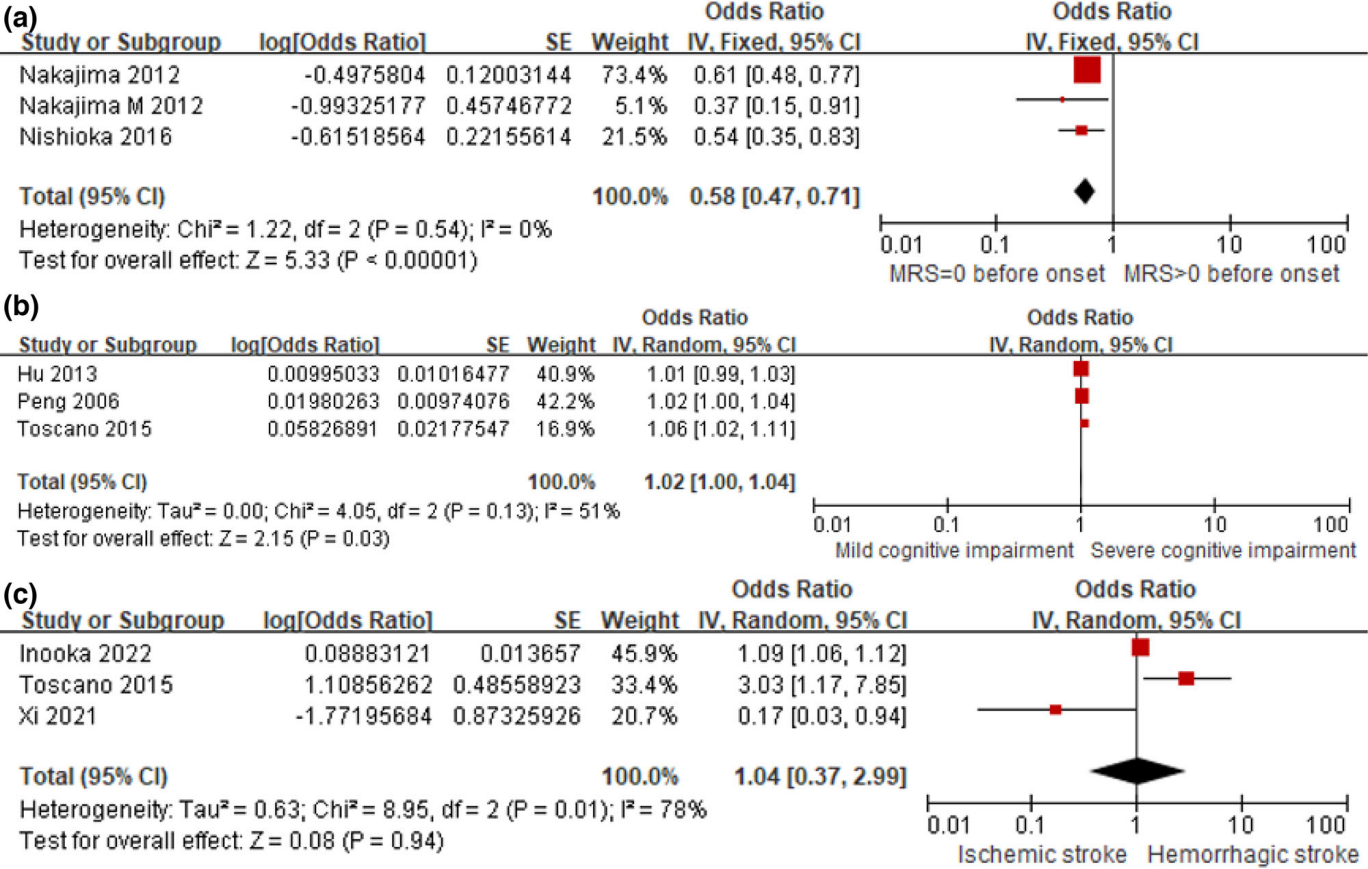
Subgroup analysis of prognostic risks. (**A**) Forest plot for mRS = 0 before onset. (**B**) Forest plot for cognitive impairment. (**C**) Forest plot for severity of stroke type.

Three studies explored showed mRS = 0 before onset was a protective factor for the prognosis of PSD, and a pooled OR of 3 studies was 0.58 (95% CI, 0.47–0.71; *I* ^2^ = 0%) (Figure 5A).

### 3.13 Cognitive impairment

Seven studies evaluated the risk of cognitive impairment on the prognosis of poststroke dysphagia and pooled analysis of 3 studies showed that severe cognitive impairment was not conducive to recovery from dysphagia of stroke patients (OR, 1.02; 95% CI, 1.00–1.04; *I* ^2^ = 51%) (Figure 5B).

### 3.14 Type of stroke

Three studies compared the effect of hemorrhagic stroke and ischemic stroke on the prognosis of PSD, and the pooled OR was 1.04 (95% CI, 0.37–2.99; *I* ^2^ = 78%). However, there was no association between stroke type and prognosis of PSD (Figure 5C).

## 4 DISCUSSION

This meta‐analysis aimed to provide a synthesis of predictive variables for the recovery of PSD. On the basis of 28 studies, we identified 12 prognostic factors allowing for pooled analysis. Compared to the previous meta‐analysis of prognostic factors for PSD that included 18 studies (Liu et al., 2022), we included 28 studies with more factors influencing the prognosis of PSD. Pooled analysis of relevant factors showed that older age, higher NIHSS score, poor ADL, lower BMI, severe dysphagia on admission, aspiration, brainstem stroke, bilateral hemispheric stroke, and severe cognitive impairment were risk factors for prognosis of PSD. mRS = 0 before onset and early intervention was promising predictor for prognosis of PSD. In addition, hemorrhagic stroke has not shown significant predictive value.

The pathogenesis of dysphagia varies between stroke sites, that is, pseudobulbar palsy and bulbar palsy. Pseudobulbar palsy is a consequence of upper motor neuron lesions caused by bilateral cortical tract disorders and is featured with dysphagia, dysphonia, face and tongue weakness, and emotional instability (Hong, 2006; McCormick & Lee, 2002). Bulbar palsy is a type of low motor neuron injury affecting the nuclei of IX, X, XI, and XII of cerebral nerves (Wang et al., 2022). The relationship between the prognosis of PSD and the stroke site has been explored in studies (Kumar et al., 2014; Wang et al., 2022). It has long been recognized that the brainstem, especially the medulla oblongata, containing the swallowing central pattern generator is of great importance in swallowing (Jean, 2001; Kessler & Jean, 1985). Previous studies have indicated that lesions in brainstem was associated with worse swallowing recovery, as the initiation and integration centers had suffered damage (Flowers et al., 2011; Jean, 2001). In line with this, our pooled meta‐analysis showed that brainstem stroke was a risk factor for the prognosis of PSD. In addition, our study identified bilateral hemispheric stroke as a negative prognostic factor for PSD. A plausible interpretation of this is that the majority of the central mechanisms involved in swallowing with bilateral input, which allow for better compensation of dysphagia in unilateral lesions (Beharry et al., 2019). Moreover, researchers believe that dysphagia may worsen if the affected side is not adequately compensated from the unaffected hemisphere (Beharry et al., 2019). Previous studies have shown inconsistent associations between stroke type and dysphagia prognosis due to differences in methodology methodology and other variables. Interestingly, our results with pooled data identifying no significant links between hemorrhagic stroke and prognosis of PSD (*p* = .94). However, given the low number of studies (*n* = 3) and heterogeneity, it is necessary to interpret our results appropriately.

Results of the present meta‐analysis showed that higher NIHSS score, lower ADL score, and severe cognitive impairment were negative predictors for the recovery of PSD. The NIHSS scale primarily assesses level of consciousness, limb motor function, sensation, facial palsy, dysarthria, and speech (Kwah & Diong, 2014). Decreased consciousness, facial palsy, and lack of facial sensation can also adversely impact swallowing function. Furthermore, speech and swallowing have overlapping anatomical structures and are thus intrinsically related processes. Notably, study has indicated that NIHSS scores in the subacute phase serve as a better prognostic factor for PSD than NIHSS scores on admission, which possibly due to the dynamics of the patient's condition in the acute phase (Nakajima et al., 2012). Regarding the relationship between cognitive impairment and dysphagia outcomes, a study showed that cognitive impairment was detrimental to the recovery of swallowing function (Castagna et al., 2019); it is likely that patients with cognitive impairment fail to actively engage in treatment, which impedes the recovery process. ADL scores potentially contain information relating to stroke severity and motor dysfunction at the time of admission; therefore, it is reasonable that lower ADL scores are associated with poorer prognosis of swallowing function.

Older age, lower BMI, severe dysphagia on admission, and aspiration are prognostic for worse PSD, similar to some previous studies (Dubin et al., 2013; Giraldo‐Cadavid et al., 2020; Lee et al., 2020). As the age increases, the strength of contraction of the orofacial muscles decreases and the response to food stimulation becomes sluggish, which affects the coordination of swallowing function. Additionally, the elderly is vulnerable to other complications, and are prone to pneumonia, malnutrition, and dehydration following swallowing disorders. Furthermore, Ahn et al. (2020) also found that older age was related to severe swallowing disorders. These may be plausible explanations for older age as a negative factor for prognosis of PSD. BMI provides an indication of the patient's overall nutritional status. Studies have previously reported that malnutrition increased the risk of complications, as well as worse clinical performance. This may account for the lower BMI being responsible for a poorer recovery of swallowing function (Wang et al., 2022). Undoubtedly, severe dysphagia at the time of admission would negatively affect nutrition intake, and further deteriorate the general function of the patient, which ultimately leads to a poorer prognosis of PSD. Aspiration represents a considerable threat to patients, which means that the risk of pneumonia tends to increase (Kosutova & Mikolka, 2021), and the occurrence of pneumonia could aggravate patients' conditions and impede their functional recovery.

Recently, some researchers considered sarcopenia as an independent risk factor for dysphagia (de Sire et al., 2021; Maeda & Akagi, 2016; Maeda et al., 2017). Sarcopenia is a syndrome that usually manifests as the loss of skeletal muscle mass and strength, which is associated with adverse outcomes (Cruz‐Jentoft et al., 2010). The muscles involved in swallowing tend to lose mass due to aging and malnutrition, and the loss of muscle mass is linked to dysphagia (Fujishima et al., 2019). Due to progressive decline in several physiological functions, the risk of sarcopenia and dysphagia in elderly people may increase (de Sire et al., 2021; Leigheb et al., 2021). In addition, Maeda et al. (2017) found a significant correlation between BMI and sarcopenic dysphagia. It should be noted that older age, poor ADL, and lower BMI have been identified as negative predictors of PSD recovery in this study, which is consistent with the above findings. In this regard, we assume that these three factors may decrease the strength and the mass of swallowing‐related muscles and thus affect the prognosis of PSD.

In the present meta‐analysis, early intervention and mRS = 0 before onset were protective factors for PSD recovery. Carnaby et al. (2006) demonstrated for the first time that early behavioral interventions combined with dietary modification did work to promote functional recovery of PSD. Similarly, Takahata et al. (2011) showed that early intervention was effective in improving swallowing and reducing the risk of lung infection in patients with acute stroke. While there were differences in study design, patient heterogeneity and assessment methods, both studies revealed that early intervention was essential for the recovery of PSD. Generally, functional recovery in stroke patients is linked to changes in neuroplasticity (Murphy & Corbett, 2009). An animal study has shown that early intervention can improve synaptic plasticity (Li et al., 2021). In addition, patients with PSD have also been observed to exhibit plasticity changes in the swallowing neural network that are responsive to the lesion and act as a critical role in the recovery of swallowing function (Cheng & Hamdy, 2022). Therefore, we consider that the prognosis of swallowing function facilitated by early intervention may be related to changes in neuroplasticity. mRS has been utilized extensively in stroke trials as a measure of premorbid capacity and outcome (Liu et al., 2020). It consists of seven levels from 0 to 6, 0 and 6 corresponding to asymptomatic and mortality, respectively (Liu et al., 2020). The higher mRS score indicates that, in addition to suffering from stroke, the patient may also have other neurological diseases that affect swallowing function (Nakajima et al., 2012). Gandolfo et al. (2019) suggested that a higher mRS score was the most reliable clinical parameters for independently predicting the persistence of dysphagia, which supports our findings.

### 4.1 Limitations

In the present meta‐analysis, an extensive search term and no restriction on country of origin were utilized to guarantee the identification of all prognostic factors for PSD. However, some limits do exist, which must be considered when interpreting the reports. First, most of the enrolled studies adopting retrospective study design introduced inherent biases, including selection, missing data, and varied duration of follow‐up. Second, this meta‐analysis of the published literature may be be subject to publication bias publication bias and restricted by the quality and methodology of the included studies. Third, due to the high considerable heterogeneity between studies, several studies were removed following sensitivity analyses, which could have affected the values reported. In addition, variables were included in the meta‐analysis only if they were reported in at least 3 publications, which potentially result in failure to assess the impact of certain important variables on the prognosis of dysphagia. Last, only articles published in English or Chinese were included in this study, and relevant studies published in other languages were not included, which may have selection bias.

## 5 CONCLUSIONS

Based on 28 studies, the present meta‐analysis summarizes 12 factors influencing the prognosis of PSD. The presented findings may have clinical utility in designing nutritional management and individualizing the treatment strategies for stroke patients with dysphagia. However, further prospective studies are still needed to investigate additional prognostic factors of PSD.

### PEER REVIEW

The peer review history for this article is available at https://publons.com/publon/10.1002/brb3.3033.

## Data Availability

The data that support the findings of this study are available from the corresponding author upon reasonable request.
